# Genome‐wide identification, expression profiling, and target gene analysis of microRNAs in the Onion thrips, *Thrips tabaci* Lindeman (Thysanoptera: Thripidae), vectors of tospoviruses (Bunyaviridae)

**DOI:** 10.1002/ece3.3762

**Published:** 2018-06-07

**Authors:** Rebijith K. Balan, Asokan Ramasamy, Ranjitha H. Hande, Suresh J. Gawande, Nallur K. Krishna Kumar

**Affiliations:** ^1^ Department of Physiology, Development, and Neuroscience University of Cambridge Cambridge UK; ^2^ Division of Biotechnology ICAR‐Indian Institute of Horticultural Research Bangalore India; ^3^ Crop Protection Section ICAR‐Directorate of Onion and Garlic Research Pune India; ^4^ Bioversity International India New Delhi India

**Keywords:** deep‐sequencing, miRNAs, sRNAs, Stem‐loop RT‐PCR, *Thrips tabaci*, tospovirus

## Abstract

*Thrips tabaci* Lindeman is an important polyphagous insect pest species estimated to cause losses of more than U.S. $1 billion worldwide annually. Chemical insecticides are of limited use in the management of *T. tabaci* due to the thigmokinetic behavior and development of resistance to insecticides. There is an urgent need to find alternative management strategies. Small noncoding RNAs (sncRNAs) especially microRNAs (miRNAs) hold great promise as key regulators of gene expression in a wide range of organisms. MiRNAs are a group of endogenously originated sncRNA known to regulate gene expression in animals, plants, and protozoans. In this study, we explored these RNAs in *T. tabaci* using deep sequencing to provide a basis for future studies of their biological and physiological roles in governing gene expression. Apart from snoRNAs and piRNAs, our study identified nine novel and 130 known miRNAs from *T. tabaci*. Functional classification of the targets for these miRNAs predicted that majority are involved in regulating transcription, translation, signal transduction and genetic information processing. The higher expression of few miRNAs (such as tta*‐*miR‐281, tta‐miR‐184, tta‐miR‐3533, tta‐miR‐N1, tta‐miR‐N7, and tta‐miR‐N9) in *T. tabaci* pupal and adult stages reflected their possible role in larval and adult development, metamorphosis, parthenogenesis, and reproduction. This is the first exploration of the miRNAome in *T. tabaci*, which not only provides insights into their possible role in insect metamorphosis, growth, and development but also offer an important resource for future pest management strategies.

## 1 INTRODUCTION

**Figure 1:**
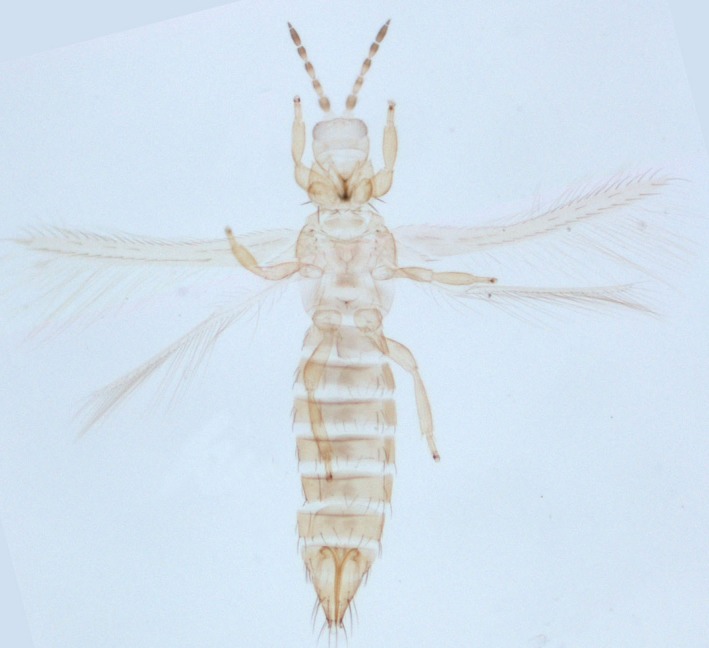
Photograph of the adult *Thrips tabaci* Lindeman, an important polyphagous insect pest species belonging to the family Photograph of the Thripidae. Image Credit : Dr. Ramaiyer Varatharajan (Manipur University, Imphal) and Rachana R R (ICAR‐ NBAIR, Bengaluru).

Onion thrips, *Thrips tabaci* Lindemann (Figure 1), is an important polyphagous insect pest species (Lewis, 1973) belonging to the family Thripidae. Besides onions, it is known to infest around 300 plant species, including economically important crops such as tobacco, leek, cabbage, pea, melon, lettuce, potato, tomato, carnation (Diaz‐Montano, Fuchs, Nault, Fail, & Shelton, 2011; Lewis, 1997; Mandal et al., 2012). *Thrips tabaci* is also a vector of two viral pathogens, *Iris yellow spot virus* (IYSV) (Srinivasan et al., 2012) and *Tomato spotted wilt virus* (TSWV) (Pittman, 1927) causing significant disease around the world (German, Ullman, & Moyer, 1992). *Thrips tabaci* is estimated to cause more than U.S. $1 billion in crop losses annually worldwide. To date, chemical insecticides have been widely used for the management of *T. tabaci*, but due to its thigmokinetic behavior and frequent development of insecticide resistance, they have had little use. Therefore, the design of novel insecticides, resistance breeding strategies, an in‐depth understanding of genes and gene regulation is necessary for targeting important developmental factors/processes for effective management of this insect. MiRNA analysis is an effective tool to understand gene regulation and expression in both insect and host plant.

MicroRNAs (miRNAs) are a group of small, sequence‐specific, endogenously originated noncoding RNA (ncRNA) molecules containing ~18–25 nucleotides (nts), and their main function is to regulate gene expression in animals, plants, and protozoans. MiRNAs controls around 60% of protein‐coding gene activities and regulates many cellular processes (Fabian, Sonenberg, & Filipowicz, 2010; Friedman, Farh, Burge, & Bartel, 2009). The function of miRNAs appears to regulate gene expression either by translation repression or by degradation of mRNA through deadenylation (Chekulaeva & Filipowicz, 2009). MiRNA‐mediated gene regulation plays a significant role in cellular and developmental processes, for instance in cell division, cell death, disease, hormone secretion, and neural development (Ambros, 2004; Miska et al., 2007; Nohata, Hanazawa, Kinoshita, Okamoto, & Seki, 2012; Singh & Nagaraju, 2008). The first miRNA, Lin‐4 gene, was discovered by Lee, Feinbaum, and Ambros (1993) in *Caenorhabditis elegans*. Consequently, several miRNAs have been discovered from wide varieties of organisms including insects (Lagos‐Quintana, Rauhut, Lendeckel, & Tuschl, 2001), plants (Bartel, 2004), viruses (Cullen, 2006), and vertebrates (Lim, Glasner, Yekta, Burge, & Bartel, 2003).

Identification of miRNA includes three principle approaches, forward genetics, bioinformatics prediction (Rebijith et al., 2014; Zhang, Pan, Cannon, Cobb, & Anderson, 2006), and direct cloning and sequencing (Chen et al., 2005; Lagos‐Quintana et al., 2001; Lee & Ambros, 2001). High‐throughput next‐generation sequencing (NGS) emerged as a powerful tool to identify miRNAs from animals and plants (Calla & Geib, 2015; Guillem, Bastian, Maria‐Dolors, & Xavier, 2016; Nandety, Sharif, Kamita, Ramasamy, & Falk, 2015; Song et al., 2011; Wang et al., 2012; Wu et al., 2013). It has accelerated the pace of miRNA discovery from various animals and plants (Avesson, Reimegard, Wagner, & Söderbom, 2012; Burnside et al., 2008; Ge et al., 2013; Hu et al., 2012; Kang et al., 2012; Koh et al., 2010; Zhang et al., 2012).

So far, the miRNAome for insects is far behind nematodes, plants, and mammals (Kakumani et al., 2015). MiRNAs are reported from about 25 species of insects belonging to various orders (Stark et al., 2007; Wu et al., 2013). No information is available on *T. tabaci* miRNA content and function. Our study reports the detailed profile of miRNAs from *T. tabaci*. Further analysis identified putative target genes for these miRNAs, which will shed more light on the identification of highly specific miRNAs for thysanopteran pest management in the near future.

## 2 MATERIALS AND METHODS

### 2.1 Insect culture and RNA isolation

*Thrips tabaci* cultures were maintained on *Phaseolus vulgaris* in controlled laboratory conditions at 25°C (DeGraaf & Wood, 2009) with an 8 hr:16 hr light:dark cycle. Total RNA was isolated from whole‐body homogenates of sample mix, containing a total of 50 mg of different life stages *viz*. eggs, larvae, pupae, and adults of *T. tabaci* using TRIzol reagent (Invitrogen, Carlsbad, CA, USA).

### 2.2 Sample preparation and Illumina sequencing

Samples were processed according to Illumina TruSeq^™^ Small RNA sample preparation guide. Size fractionated small RNA populations (18–28 nts) were extracted, purified, and ligated to 3′ and 5′ adapters using T4 RNA Ligase (Life Technologies, Ambion, USA). Ligated products were reverse transcribed using SuperScript II (Life Technologies, Invitrogen, USA) followed by PCR amplification with 11 cycles and two size selection gels. High‐throughput sequencing of the small RNA libraries was performed on Illumina Hiseq2000.

### 2.3 Bioinformatics analysis of small RNA sequencing data

The obtained sequenced dataset was subjected to initial quality check, and the raw reads were taken for adapter trimming and filtering of low‐quality data. Thus, obtained sequencing data were queried against Rfam (http://rfam.sanger.ac.uk/) and RepBase (http://www.girinst.org/repbase/) as references to annotate the ncRNAs *viz*. rRNAs, tRNAs, snRNAs, snoRNAs, and repeat‐associated small RNAs and degraded fragments of expressed genes (exons and introns) in the remaining sequences. Remaining unique sequences were aligned with the miRBase (v21, http://www.miRBase.org/) entries to identify the conserved miRNAs. Novel miRNAs and their star reads were identified using the miRDeep2 (Friedlander, Mackowiak, Li, Chen, & Rajewsky, 2012) and miRCat (http://srna-workbench.cmp.uea.ac.uk/tools/mircat/). Potential secondary hairpin structures for identified novel miRNAs were predicted by employing Mfold (http://mfold.rna.albany.edu/?q_mfold/RNA-folding-form).

Homology analysis was performed with conserved miRNAs of *T. tabaci* with the miRNAs of other organisms from the miRBase database (Release 21.0; Griffiths‐Jones, Saini, van Dongen, & Enright, 2008). BLASTn embedded in the miRBase database was used to compare the *T. tabaci* miRNAs with other species, with an *E*‐value of .01 to find out more miRNA homologs. The naming of the miRNAs in this study has been performed according to Griffiths‐Jones, Grocock, van Dongen, Bateman, & Enright, 2006. As these miRNAs were predicted from *T. tabaci,* the prefix for all miRNAs was fixed as “tta.” The rest of the naming convention criteria were in accordance with miRBase (Griffiths‐Jones et al., 2006).

### 2.4 Phylogenetic analysis of microRNA family

All the identified miRNAs were classified into different miRNA precursor families (http://www.rfam.sanger.ac.uk), and primary sequence analyses were performed by employing Bioedit (Hall, 1999) and Weblogo (http://weblogo.berkeley.edu/logo.cgi). Few miRNA families such as miR‐8, miR‐14, miR‐276, and miR‐281 were selected for phylogenetic analysis employing RaxML.v.7.0.4 (Stamatakis, 2008).

### 2.5 Target prediction

Targets for identified miRNAs were predicted employing the miRanda program (Enright et al., 2004), against the expressed sequence tags (ESTs) and transcriptome (NCBI Accession: PRJNA203209) database of *Frankliniella occidentalis*. An alignment score (Smith & Waterman, 1981) greater than or equal to 100 and miRNA:mRNA Minimum Free Energy (MFE, ∆G) less than −20 kcal/mol were considered as putative target genes. The targets were further annotated against NCBI‐RefSeq invertebrate protein database and Gene Ontology (GO) terms were assigned (using Blast‐2‐GO) based on the annotation. The circos plot was generated using Circos (Krzywinski et al., 2009) to visualize the interaction between miRNAs and their targets.

### 2.6 Validation of *Thrips tabaci* miRNAs using Stem‐loop RT‐PCR

We were able to validate six conserved and four novel microRNAs employing Stem‐loop RT‐PCR primers designed based on previous reports (Chen et al., 2005).

### 2.7 Differential expression of *Thrips tabaci* miRNAs using Quantitative Real‐Time PCR

Differentially expressed and functionally significant ten miRNAs (six conserved and four novel) were selected for quantitative reverse transcriptase PCR (qRT‐PCR). Total RNA was isolated from different life stages *viz*. larvae, pupae, and adults of *T. tabaci* using TRIzol reagent (Invitrogen, Carlsbad, CA, USA). Mir‐X‐miRNA qRT‐PCR SYBR Kit (Clontech Laboratories, Inc., USA) was used for the qRT‐PCR reactions. qRT‐PCR was performed on Light Cycler 480 (Roche, USA) using 1:20 diluted cDNAs and SYBR Advantage Premix (Clontech Laboratories, Mountain View, USA), according to the manufacturer's instructions. All the qRT‐PCR assays were conducted according to the MIQE guidelines (Bustin et al., 2009). qRT‐PCR assays were performed in triplicates for three independent biological replicates, and the relative gene expression data were analyzed using $2^{-\Delta \Delta C_{T}}$ method (Livak & Schmittgen, 2001). U6 snRNAs was used as an internal control gene for normalization. The values of these three independent experiments were statistically analyzed using one‐way ANOVA to calculate the statistical significance.

## 3 RESULTS

### 3.1 Illumina sequencing of *Thrips tabaci* small RNAs

**Figure 2:**
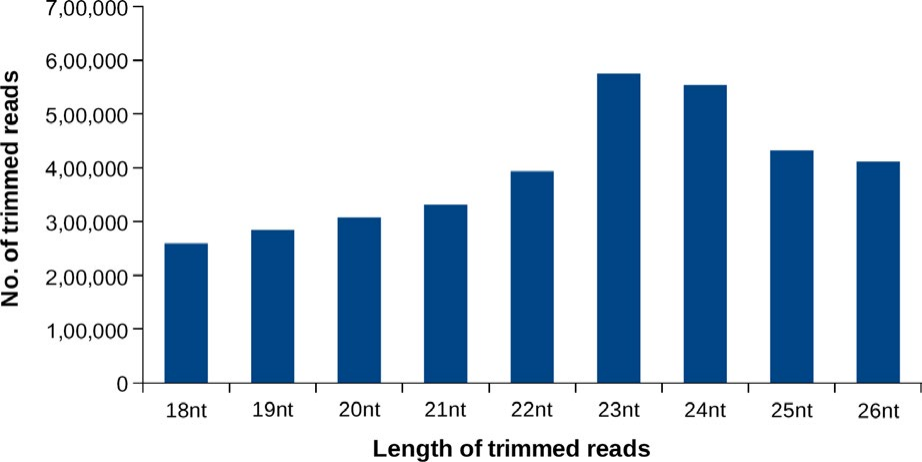
Length distribution of mappable reads (≥18 nt to ≤26 nt) obtained from *Thrips tabaci* deep sequencing

The small RNA library prepared for deep sequencing resulted in a total of 13,192,454 raw reads (Table 1). After various mapping (Table 1), the trimmed high‐quality small RNA reads were employed to identify both known and novel miRNAs. Size distributions of the trimmed high‐quality reads were varied from 18 to 26 nts with a peak at the 23 nts (Figure 2). A small portion of our library consisted of read length of around 26–28 nts, which could be putative piwi‐interacting RNAs (piRNAs) from *T. tabaci* as the homology search against the piRNABank database revealed that some of these were similar to previously reported piRNAs (Table 2).

**Table 1:** Summary Statistics of *Thrips tabaci* small RNA data analysis

Number of trimmed reads	13,192,454
Mapped to mRNA	2,378,671
Repbase mapped reads	1,396,829
Rfam mapped reads	4,181,894
Rfam unmapped reads	9,010,560
miRBase mapped reads	47,570
Total unmappable for miRNA	5,187,490
Average length	23

**Table 2:** Small RNAs (Piwi RNAs) with nucleotide lengths larger than 25 nucleotides obtained from *Thrips tabaci* sequencing data

smallRNA ID	Sequence (5′‐>3′	Length (nt)	Hit in the piRNABank	*E*‐value
tta_piR1	ATTGTGGTTCAGTGGTAGAATTCTCGCC	28	hsa_piR_018570	.00065
tta_piR2	GGGTTCGATTCCCGGTCAGGGAACCA	26	dr_piR_0017650	.1
tta_piR3	TTTCCGTAGTGTAGTGGTTATCACGTTC	28	rno_piR_005901	1.70E‐05
tta_piR4	CCAAAGCAUCGCGAAGGCCCACGGCG	26	dr_piR_0052831	.0047
tta_piR5	ATTGGTGGTTCAGTGGTAGAATTCTCGC	28	hsa_piR_001312	1.80E‐05
tta_piR6	CCCTCGGTTCTGGCGTCAAGCGGGCCG	27	No Hit	NA
tta_piR7	CCTGTGGTCTAGTGGTTAGGATTCGGCG	28	ona_piR_166322	.00049

### 3.2 Identification of known miRNAs from *Thrips tabaci*

Our analyses on the trimmed high‐quality reads resulted in a total of 130 conserved miRNAs representing 55 different miRNA families (Table 3). Among the known miRNAs, miR‐276, miR‐281, miR‐8, and miR‐14 are highly expressed with an expression value of 26,418, 18,063, 16,204, and 12,453, respectively (Table 3). Analysis of the 55 miRNA families revealed that most of them were present in arthropod species (Table 4), with many homologous miRNAs from *Aedes aegypti*,* Apis mellifera*,* Bombyx mori*,* Acyrthosiphon pisum,* and *Tribolium castaneum* (Figure S1).

**Table 3:** Expression value of known miRNAs in *Thrips tabaci*. The first column represents miRNA family; the second column represents the number of reads annotated on the particular miRNA family; the third column represents length of the mature miRNA sequences; the fourth column represents the name of the miRNAs in *T. tabaci*; the fifth column represents the miRNA sequence; the sixth column represents homologous species of organism where it has the highest similarity

miRNA family	Expression values^a^ (Reads)	Length (nt)	Name of the miRNA	Sequence (5′–3′)	Resource
mir‐281	468	19	tta‐miR‐281a	AAGAGAGCUAUCCGUCGAC	*Aedes aegypti*
mir‐281	17583	22	tta‐miR‐281b	AAGAGAGCUAUCCGUCGACAGU	*Bombyx mori*
mir‐281	4	22	tta‐miR‐281c	AAGAGAGCUGUCCGUCGACAGU	*Drosophila ananassae*
mir‐281	5	21	tta‐miR‐281d	AAGGGAGCAUCUGUCGACAGU	*Lottia gigantea*
mir‐281	3	22	tta‐miR‐281e	UGUCAUGGAGUUGCUCUCUUUU	*Branchiostoma belcheri*
mir‐276	507	21	tta‐miR‐276a	UAGGAACUUCAUACCGUGCUC	*Aedes aegypti*
mir‐276	25904	22	tta‐miR‐276b	UAGGAACUUCAUACCGUGCUCU	*Locusta migratoria*
mir‐276	7	22	tta‐miR‐276c	UAGGAACUUAAUACCGUGCUCU	*Drosophila ananassae*
mir‐306	173	21	tta‐miR‐306a	UCAGGUACUAGGUGACUCUGA	*Bombyx mori*
mir‐306	3507	22	tta‐miR‐306b	UCAGGUACUGAGUGACUCUGAG	*Apis mellifera*
mir‐306	1976	22	tta‐miR‐306c	UCAGGUACUGAGUGACUCUCAG	*Aedes aegypti*
bantam	61	21	tta‐miR‐bantam‐a	UGAGAUCAUUGUGAAAGCUAU	*Brugia malayi*
bantam	33	22	tta‐miR‐bantam‐b	UGAGAUCAUUUUGAAAGCUGAU	*Aedes aegypti*
bantam	1889	23	tta‐miR‐bantam‐c	UGAGAUCAUUGUGAAAGCUGAUU	*Apis mellifera*
bantam	5	23	tta‐miR‐bantam‐d	UGAGAUCAUUGUGAAAGCUAAUU	*Acyrthosiphon pisum*
mir‐92	5	20	tta‐miR‐92a	UAUUGCACUCGUCCCGGCCU	*Brugia malayi*
mir‐92	8	22	tta‐miR‐92b	UAUUGCACCAGUCCCGGCCUAU	*Bombyx mori*
mir‐92	6	22	tta‐miR‐92c	UAUUGCACCUGUCCCGGCCGAU	*Ciona savignyi*
mir‐92	76	22	tta‐miR‐92d	UAUUGCACUCGUCCCGGCCUUG	*Oikopleura dioica*
mir‐92	4	23	tta‐miR‐92e	UAUUGCACCAGUCCCGGCCUGAC	*Tribolium castaneum*
mir‐92	1861	22	tta‐miR‐92f	UAUUGCACUCGUCCCGGCCUGU	*Saccoglossus kowalevskii*
mir‐92	629	22	tta‐miR‐92 g	UAUUGCACUCGUCCCGGCCUGC	*Lytechinus variegatus*
mir‐92	46	22	tta‐miR‐92 h	AAUUGCACCCGUCCCGGCCUGA	*Apis mellifera*
mir‐750	4	22	tta‐miR‐750a	CAGAUCUAACUCUUCCAGCUCA	*Lottia gigantea*
mir‐750	1242	22	tta‐miR‐750b	CCAGAUCUAACUCUUCCAGCUC	*Apis mellifera*
mir‐750	107	23	tta‐miR‐750c	CCAGAUCUAACUCUUCCAGCUCA	*Capitella teleta*
mir‐10	433	21	tta‐miR‐10a	ACCCUGUAGAUCCGAAUUUGU	*Acyrthosiphon pisum*
mir‐10	6	21	tta‐miR‐10b	UACCCUGUAGAUCCGAAUUUG	*Ovis aries*
mir‐10	4	22	tta‐miR‐10c	UACCCUGUAGAACCGAAUUUGU	*Anolis carolinensis*
mir‐10	6	22	tta‐miR‐10d	ACCCUGUAGAUCCGAAUUUGUU	*Aedes aegypti*
mir‐10	9	22	tta‐miR‐10e	AACCCUGUAGACCCGAAUUUGA	*Gyrodactylus salaris*
mir‐10	52	22	tta‐miR‐10f	UACCCUGUAGAUCCGAAUUUGU	*Lottia gigantea*
mir‐10	3	23	tta‐miR‐10 g	UACCCUGUAGAACCGAAUUUGUG	*Bos taurus*
mir‐10	16	23	tta‐miR‐10 h	UACCCUGUAGAAUCGAAUUUGUG	*Anolis carolinensis*
mir‐10	3	23	tta‐miR‐10i	AACCCUGUAGAUCCGAGUUAGAU	*Schmidtea mediterranea*
mir‐100	5	21	tta‐miR‐100a	AACCCGUAGAUCCGAACUUGU	*Capra hircus*
mir‐100	20	22	tta‐miR‐100b	AACCCGUAGAUCCGAACUUGUG	*Ateles geoffroyi*
mir‐100	57	23	tta‐miR‐100c	AACCCGUAGAUCCGAACUUGUGU	*Branchiostoma floridae*
mir‐100	3	24	tta‐miR‐100d	AACCCGUAGAUCCGAACUUGUGUU	*Ascaris suum*
mir‐1000	11	18	tta‐miR‐1000a	AUAUUGUCCUGUCACAGC	*Tribolium castaneum*
mir‐1000	192	21	tta‐miR‐1000b	AUAUUGUCCUGUCACAGCAGU	*Drosophila melanogaster*
mir‐1000	183	22	tta‐miR‐1000c	AUAUUGUCCUGUCACAGCAGUA	*Drosophila pseudoobscura*
mir‐8	248	22	tta‐miR‐8a	UAAUACUGUCAGGUAAAGAUGU	*Culex quinquefasciatus*
mir‐8	15951	23	tta‐miR‐8b	UAAUACUGUCAGGUAAAGAUGUC	*Capitella teleta*
mir‐8	5	22	tta‐miR‐8c	CAUCUUACCGGGCAGCAUUAGA	*Aedes aegypti*
mir‐9	8	18	tta‐miR‐9a	UCUUUGGUAUCCUAGCUG	*Bombyx mori*
mir‐9	7	21	tta‐miR‐9b	UCUUUGGUGAUCUAGUUGUAU	*Tribolium castaneum*
mir‐9	6	21	tta‐miR‐9c	UCUUUGGUACUUUAGCUGUAG	*Acyrthosiphon pisum*
mir‐9	13	23	tta‐miR‐9d	UCUUUGGUUAUCUAGCUGUAUGA	*Capitella teleta*
mir‐9	4	24	tta‐miR‐9e	UCUUUGGUUUUCUAGCUGUAUGAU	*Schmidtea mediterranea*
mir‐2	21	23	tta‐miR‐2a	UAUCACAGCCAGCUUUGAUGAGC	*Apis mellifera*
mir‐2	27	23	tta‐miR‐2b	UAUCACAGCCAGCUUUGAUGAGU	*Lottia gigantea*
mir‐2	27	24	tta‐miR‐2c	UAUCACAGCCAGCUUUGAUGAGCU	*Aedes aegypti*
mir‐184	7145	21	tta‐miR‐184a	UGGACGGAGAACUGAUAAGGG	*Anopheles gambiae*
mir‐184	142	22	tta‐miR‐184b	UGGACGGAGAACUGAUAAGGGU	*Anolis carolinensis*
mir‐184	118	22	tta‐miR‐184c	UGGACGGAGAACUGAUAAGGGC	*Ixodes scapularis*
mir‐279	20	21	tta‐miR‐279a	UGACUAGAUCCACACUCAUCC	*Acyrthosiphon pisum*
mir‐279	58	22	tta‐miR‐279b	UGACUAGAUCCACACUCAUCCA	*Lottia gigantea*
mir‐279	14	22	tta‐miR‐279c	UGACUAGAUCCACACUCAUUAA	*Anopheles gambiae*
mir‐279	4	22	tta‐miR‐279d	UGACUAGAUCUACACUCAUUGA	*Bombyx mori*
mir‐279	105	22	tta‐miR‐279e	UGACUAGAGUCACACUCGUCCA	*Apis mellifera*
mir‐279	635	22	tta‐miR‐279f	UGACUAGAUCCAUACUCGUCUG	*Bombyx mori*
mir‐279	26	24	tta‐miR‐279 g	UGACUAGAUCGAAAUACUCGUCCC	*Apis mellifera*
mir‐279	103	25	tta‐miR‐279 h	UGACUAGAUCCAUACUCGUCUAUAG	*Tribolium castaneum*
mir‐2796	65	21	tta‐miR‐2796a	AGGCCGGCGGAAACUACUUGC	*Nasonia vitripennis*
mir‐2796	5	22	tta‐miR‐2796b	GUAGGCCGGCGGAAACUACUAG	*Acyrthosiphon pisum*
mir‐2796	168	23	tta‐miR‐2796c	GUAGGCCGGCGGAAACUACUUGC	*Apis mellifera*
mir‐14	26	21	tta‐miR‐14a	UCAGUCUUUUUCUCUCUCCUA	*Anopheles gambiae*
mir‐14	12427	22	tta‐miR‐14b	UCAGUCUUUUUCUCUCUCCUAU	*Acyrthosiphon pisum*
mir‐993	3	20	tta‐miR‐993a	UACCCUGUAGAUCCGGGCUU	*Tribolium castaneum*
mir‐993	110	23	tta‐miR‐993b	GAAGCUCGUCUCUACAGGUAUCU	*Acyrthosiphon pisum*
mir‐993	10	23	tta‐miR‐993c	UACCCUGUAGAUCCGGGCUUUUG	*Manduca sexta*
mir‐993	3	23	tta‐miR‐993d	UACCCUGUAGUUCCGGGCUUUUG	*Drosophila melanogaster*
mir‐1175	106	23	tta‐miR‐1175a	AAGUGGAGCAGUGGUCUCUUCAC	*Tribolium castaneum*
mir‐1175	17	22	tta‐miR‐1175b	AAGUGGAGUAGUGGUCUCAUCG	*Aedes aegypti*
mir‐1175	4	23	tta‐miR‐1175c	UGAGAUUCACUCCUCCAACUUAC	*Apis mellifera*
mir‐1175	56	24	tta‐miR‐1175d	UGAGAUUCAACUCCUCCAACUUAA	*Bombyx mori*
mir‐124	106	21	tta‐miR‐124a	UAAGGCACGCGGUGAAUGCCA	*Schmidtea mediterranea*
mir‐124	84	21	tta‐miR‐124b	UAAGGCACGCGGUGAAUGCUA	*Anolis carolinensis*
mir‐263	4	21	tta‐miR‐263a	AAUGGCACUGGAAGAAUUCAC	*Bombyx mori*
mir‐263	18	23	tta‐miR‐263b	AAUGGCACUGGAAGAAUUCACGG	*Aedes aegypti*
mir‐263	20	24	tta‐miR‐263c	AAUGGCACUGGAAGAAUUCACGGG	*Drosophila melanogaster*
mir‐2944	18	22	tta‐miR‐2944a	UAUCACAGCAGUAGUUACCUGA	*Aedes aegypti*
mir‐2944	13	23	tta‐miR‐2944b	UAUCACAGCAGUAGUUACCUGGU	*Apis mellifera*
mir‐13	399	22	tta‐miR‐13a	UAUCACAGCCACUUUGAUGAGC	*Tribolium castaneum*
mir‐13	17	23	tta‐miR‐13b	UAUCACAGCCAUUUUUGACGAGU	*Bombyx mori*
mir‐34	15	22	tta‐miR‐34a	UGGCAGUGUGGUUAGCUGGUUG	*Aedes aegypti*
mir‐34	5	23	tta‐miR‐34b	UGGCAGUGUGGUUAGCUGGUUGU	*Ascaris suum*
mir‐34	3	23	tta‐miR‐34c	UGGCAGUGUGGUUAGCUGGUAGU	*Lottia gigantea*
mir‐133	15	22	tta‐miR‐133a	UUGGUCCCCGUCAACCAGCUGU	*Schmidtea mediterranea*
mir‐133	14	22	tta‐miR‐133b	UUGGUCCCCUUCAACCAGCUGU	*Drosophila persimilis*
mir‐317	5	21	tta‐miR‐317a	UGAACACAGCUGGUGGUAUCU	*Acyrthosiphon pisum*
mir‐317	13	24	tta‐miR‐317b	UGAACACAGCUGGUGGUAUCUUCU	*Lottia gigantea*
mir‐317	13	25	tta‐miR‐317c	UGAACACAGCUGGUGGUAUCUCAGU	*Apis mellifera*
mir‐317	4	25	tta‐miR‐317d	UGAACACAGCUGGUGGUAUCUCUUU	*Capitella teleta*
mir‐12	13	21	tta‐miR‐12a	UGAGUAUUACAUCAGGUACUG	*Tribolium castaneum*
mir‐12	3	23	tta‐miR‐12b	UGAGUAUUACAUCAGGUACUGGU	*Daphnia pulex*
mir‐252	4	22	tta‐miR‐252a	CUAAGUACUAGUGCCGCAGGAG	*Drosophila melanogaster*
mir‐252	5	23	tta‐miR‐252b	CUAAGUACUAGUGCCGCAGGAGU	*Saccoglossus kowalevskii*
mir‐277	11	22	tta‐miR‐277a	UAAAUGCACUAUCUGGUACGAC	*Aedes aegypti*
mir‐277	5	23	tta‐miR‐277b	UAAAUGCACUAUCUGGUACGACA	*Acyrthosiphon pisum*
mir‐31	3	21	tta‐miR‐31a	AGGCAAGAUGUCGGCAUAGCU	*Tribolium castaneum*
mir‐31	7	22	tta‐miR‐31b	GGCAAGAUGUCGGCAUAGCUGA	*Apis mellifera*
mir‐3477	69	23	tta‐miR‐3477a	UAAUCUCAUGCGGUAACUGUGAG	*Apis mellifera*
mir‐3477	121	22	tta‐miR‐3477b	UAAUCUCAUGUGGUAACUGUGA	*Apis mellifera*
mir‐2779	5	20	tta‐miR‐2779	AUAUCCGGCUCGAAGGACCA	*Bombyx mori*
mir‐929	4	22	tta‐miR‐929	AAAUUGACUCUAGUAGGGAGUC	*Drosophila melanogaster*
mir‐71	172	22	tta‐miR‐71	UCUCACUACCUUGUCUUUCAUG	*Tribolium castaneum*
mir‐375	4	22	tta‐miR‐375	UUUGUUCGUUCGGCUCGAGUUA	*Apis mellifera*
mir‐190	3	24	tta‐miR‐190	AGAUAUGUUUGAUAUUCUUGGUUG	*Acyrthosiphon pisum*
mir‐7550	3	18	tta‐miR‐7550	AUCCGGCUCGAAGGACCA	*Ictalurus punctatus*
mir‐482	3	22	tta‐miR‐482	GGAAUGGGCUGAUUGGGAAGCA	*Phaseolus vulgaris*
mir‐2478	3	20	tta‐miR‐2478	GUAUCCCACUUCUGACACCA	*Bos taurus*
mir‐316	3	21	tta‐miR‐316	UGUCUUUUUCCGCUUUGCUGC	*Heliconius melpomene*
mir‐3049	98	23	tta‐miR‐3049	UCGGGAAGGUAGUUGCGGCGGAU	*Apis mellifera*
mir‐996	57	21	tta‐miR‐996	UGACUAGAUACAUACUCGUCU	*Apis mellifera*
mir‐275	40	23	tta‐miR‐275	UCAGGUACCUGAAGUAGCGCGCG	*Anopheles gambiae*
mir‐965	31	22	tta‐miR‐965	UAAGCGUAUAGCUUUUCCCCUU	*Tribolium castaneum*
mir‐67	25	24	tta‐miR‐67	UCACAACCUCCUUGAGUGAGUUGA	*Ascaris suum*
mir‐315	21	23	tta‐miR‐315	UUUUGAUUGUUGCUCAGAAAGCC	*Acyrthosiphon pisum*
mir‐305	14	23	tta‐miR‐305	UUUGUACUUCAUCAGGUGCUCUG	*Tetranychus urticae*
mir‐894	11	20	tta‐miR‐894	CGUUUCACGUCGGGUUCACC	*Physcomitrella patens*
mir‐3533	9	20	tta‐miR‐3533	AUGAAGUGUGACGUGGACAU	*Bos taurus*
mir‐307	9	20	tta‐miR‐307	UCACAACCUCCUUGAGUGAG	*Daphnia pulex*
mir‐2765	664	22	tta‐miR‐2765	UGGUAACUCCACCACCGUUGGC	*Bombyx mori*
mir‐210	22	21	tta‐miR‐210	CUUGUGCGUGUGACAGCGGCU	*Drosophila melanogaster*
mir‐1	650	22	tta‐miR‐1	UGGAAUGUAAAGAAGUAUGGAG	*Drosophila melanogaster*
mir‐87	18	21	tta‐miR‐87	GUGAGCAAAGUUUCAGGUGUG	*Ixodes scapularis*
let‐7	279	21	tta‐let‐7	TGAGGTAGTAGGTTGTATAGT	*Drosophila melanogaster*
mir‐3791	15	21	tta‐miR‐3791	UCACCGGGUAGGAUUCAUCCA	*Apis mellifera*
Plant‐specific miRNA					
mir‐9774	6	22	–	CAAGATATTGGGTATTTCTGTC	*Triticum aestivum*

a Expression value is equivalent to number of miRNA reads from the library.

**Table 4:** Homology analysis of *Thrips tabaci* miRNA homologs

tta‐miR	Insects	Other Arthropods	Other Invertbrates	Vertebrates	Note
tta‐bantam	√	√	√	—	Invertebrate specific
tta‐let‐7	√	√	√	√	Highly conserved
tta‐miR‐1	√	—	—	—	Insect specific
tta‐miR‐10	√	√	√	√	Highly conserved
tta‐miR‐100	√	√	√	√	Highly conserved
tta‐miR‐1000	√	—	—	—	Insect specific
tta‐miR‐1175	√	—	√	—	Invertebrate specific
tta‐miR‐12	√	—	—	—	Insect specific
tta‐miR‐124	√	√	√	√	Highly conserved
tta‐miR‐13	√	—	—	—	Insect specific
tta‐miR‐133	√	√	√	√	Highly conserved
tta‐miR‐14	√	—	—	—	Insect specific
tta‐miR‐184	√	√	√	√	Highly conserved
tta‐miR‐190	√	—	√	√	Highly conserved
tta‐miR‐2	√	√	√	—	Invertebrate specific
tta‐miR‐210	√	—	√	√	Highly conserved
tta‐miR‐2478	—	—	—	√	Vertebrate specific
tta‐miR‐252	√	—	√	—	Invertebrate specific
tta‐miR‐263	√	√	√	—	Invertebrate specific
tta‐miR‐275	√	√	—	—	Arthropod specific
tta‐miR‐276	√	√	—	—	Arthropod specific
tta‐miR‐2765	√	—	—	—	Insect specific
tta‐miR‐277	√	—	—	—	Insect specific
tta‐miR‐2779	√	—	—	—	Insect specific
tta‐miR‐279	√	√	√	—	Invertebrate specific
tta‐miR‐2796	√	—	—	—	Insect specific
tta‐miR‐281	√	√	√	√	Highly conserved
tta‐miR‐2944	√	—	—	—	Insect specific
tta‐miR‐3049	√	—	—	—	Insect specific
tta‐miR‐305	√	√	—	—	Arthropod specific
tta‐miR‐306	√	√	—	—	Arthropod specific
tta‐miR‐307	√	√	√	—	Invertebrate specific
tta‐miR‐31	√	—	—	—	Insect specific
tta‐miR‐315	√	√	√	√	Highly conserved
tta‐miR‐316	√	√	—	—	Arthropod specific
tta‐miR‐317	√	√	√	—	Invertebrate specific
tta‐miR‐34	√	√	√	—	Invertebrate specific
tta‐miR‐3477	√	—	—	—	Insect specific
tta‐miR‐3533	—	—	—	√	Vertebrate specific
tta‐miR‐375	√	√	√	—	Invertebrate specific
tta‐miR‐3791	√	—	—	—	Insect specific
tta‐miR‐482	—	—	√	—	Invertebrate specific
tta‐miR‐67	—	—	√	—	Invertebrate specific
tta‐miR‐71	—	—	√	—	Invertebrate specific
tta‐miR‐750	√	—	—	—	Insect specific
tta‐miR‐7550	—	—	—	√	Vertebrate specific
tta‐miR‐8	√	√	√	—	Invertebrate specific
tta‐miR‐87	√	—	—	—	Insect specific
tta‐miR‐894	—	—	—	√	Vertebrate specific
tta‐miR‐9	√	√	√	√	Highly conserved
tta‐miR‐92	√	√	√	√	Highly conserved
tta‐miR‐929	√	√	√	√	Highly conserved
tta‐miR‐965	√	√	—	—	Arthropod specific
tta‐miR‐993	√	√	√	—	Invertebrate specific
tta‐miR‐996	√	—	—	—	Insect specific

### 3.3 Identification of novel miRNAs from *Thrips tabaci*

**Figure 3:**
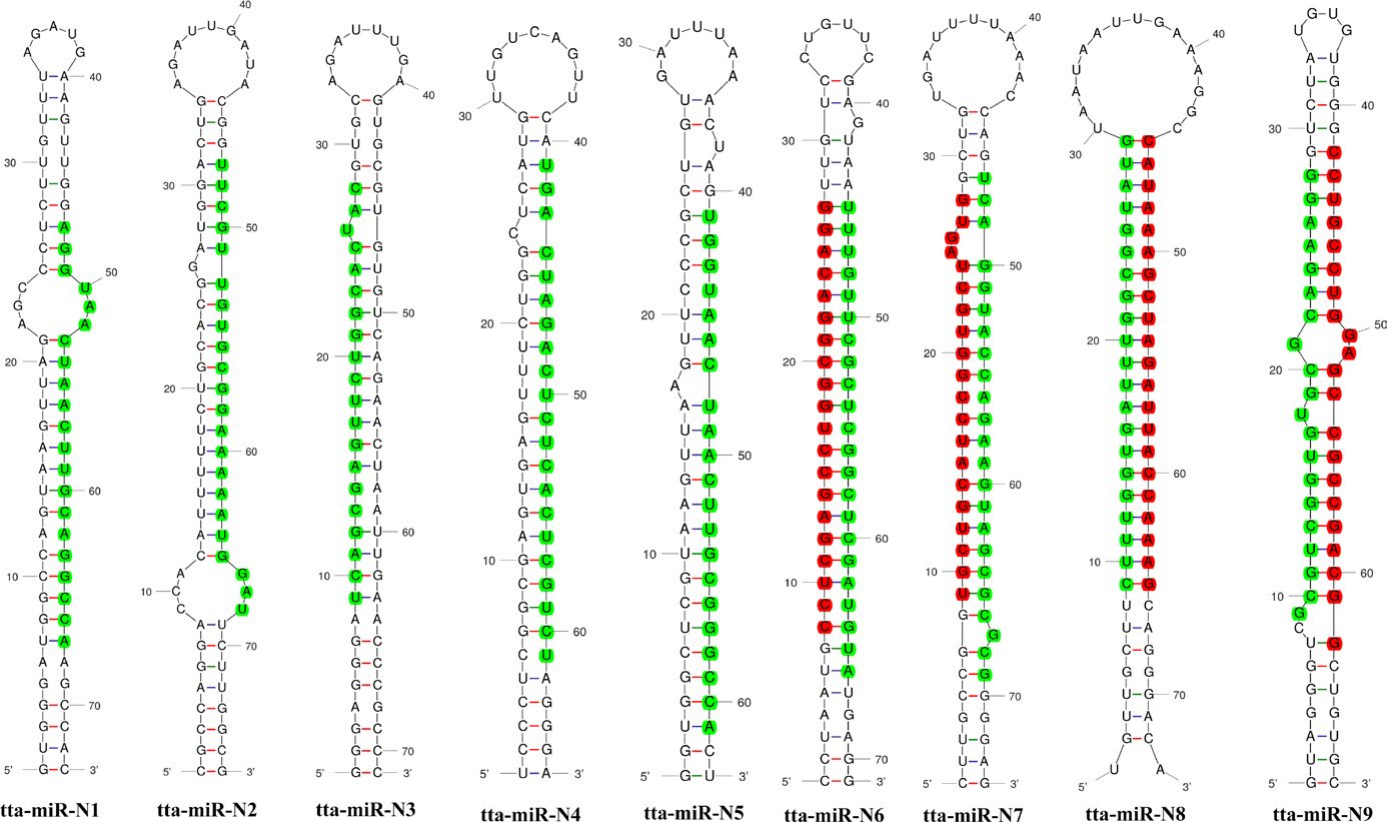
Stem‐loop structures of nine novel *Thrips tabaci* miRNAs indicating mature miRNA sequence (green color) and miRNA star strand sequences (red color)

Miranalyzer pipeline identified a total of nine novel miRNAs from *T. tabaci* for the first time (Table 5), with their predicted precursor secondary structures (Figure 3). The complete details of the mature miRNAs and their corresponding pre‐miRNAs have been given in Table 5. The length of the novel miRNAs ranged from 21 to 22 nucleotides with a preference of Uracil (66.7%) followed by Adenine (22.2%) at the 5′ end. The length of the pre‐miRNAs was in the range of 63–76 nucleotides with an average Minimum Free Energy (MFE) of −35.97 kcal/mol, indicating pre‐miRNAs are readily folded into their secondary structures. Among these nine miRNAs, three were located in the 5′ arm while the other six arose from 3′ arm (Table 5, Figure 3). tta‐miR‐N4 (3414 copies) and tta‐miR‐N7 (1978 copies) were having the highest abundance compared to the remaining novel miRNAs (Table 5).

**Table 5:** Details of *Thrips tabaci* novel miRNAs and its star strands obtained from this study. Information regarding mature, star and precursor sequences, start and end position, orientation, expression values, MFE value and (A+U) content, etc. have been given

MicroRNA Family	Name of the Novel miRNA	miRNA sequence	miRNA* sequence	Hairpin sequence	Start	End	Orientation	miRNA Reads	miRNA* Reads	MFE
Novel miRNA‐1	tta‐miR‐N1	AGGUAACUAACUUGCAGGCCA	NO	GUGGGAUGGCCAGUAAGUUAGAGCCCUCUUGUUUAGAUGAAGUUGGAGGUAACUAACUUGCAGGCCAAGCCAC	3630	3650	‐ [Negative strand]	28	Nil	−32.9
Novel miRNA‐2	tta‐miR‐N2	UUCGUUGUGCGGAAAAAUGGAU	NO	CGCCAGGACCACAUUUUUCUGCACGGAUGGACUGAGAUUGAUACGGUUCGUUGUGCGGAAAAAUGGAUUCUUGGCG	1439	1460	+ [Positive strand]	6	Nil	−40
Novel miRNA‐3	tta‐miR‐N3	AUCAGCGAGUUCUGGCACUAC	NO	GGGAGGGAUCAGCGAGUUCUGGCACUACGUGCAGAUUUGAGUGCGUGUGUCAGAACUAAUUGAACCCGCCC	11956	11976	+ [Positive strand]	15	Nil	−39.6
Novel miRNA‐4	tta‐miR‐N4	UGACUAGACUCUCACUCGUCU	NO	UCCCUCGGCGAGUGAGUUUCUGGCUCAUGUUGUCAGUUCAUGACUAGACUCUCACUCGUCUAGGGA	8647	8667	+ [Positive strand]	3414	Nil	−37.2
Novel miRNA‐5	tta‐miR‐N5	UGGUAACUAACUUGCGGGCCA	NO	GGUGGCUCGUAAGUUAAGUUCCCGCUGUGAUUUAAACUAGUGGUAACUAACUUGCGGGCCACU	13477	13497	‐ [Negative strand]	305	Nil	−35.1
Novel miRNA‐6	tta‐miR‐N6	UUUGUUCGCUCGGCUCGAUGUA	CCUCGAGCCUGGCGGACAGGU(5) CCUCGAGCCUGGCGGACAGGUU(9)	CCUAAUGCCUCGAGCCUGGCGGACAGGUUGUCCUGUUCGAGUAAUUUGUUCGCUCGGCUCGAUGUAUGAGG	1925	1946	+ [Positive strand]	528	14	−37.9
Novel miRNA‐7	tta‐miR‐N7	UCAGGUACCAGAAGUAGCGCG	GUGCUGCAUCCGGUGCUAGUG(1)	CUUGCCGGUGCUGCAUCCGGUGCUAGUGGCUGUGAUUUUAAACCAGUCAGGUACCAGAAGUAGCGCGCGGGGAG	12973	12993	‐ [Negative strand]	1978	1	−36
Novel miRNA‐8	tta‐miR‐N8	UCUUUGGUGAUUUGGCGGUAUG	AUAAAGCUAGAUUACCAAAGC(6) AUAAAGCUAGAUUACCAAAGCA(6)	UGUUGCUUCUUUGGUGAUUUGGCGGUAUGUAAUAAUUGAAAGGCCAUAAAGCUAGAUUACCAAAGCAGGGACA	14289	14310	+ [Positive strand]	22	12	−29.3
Novel miRNA‐9	tta‐miR‐N9	CGCGUCGGUGUGCGCAGAAGG	CCUGCCUGGAGCCGCCGACGG(3)	GUAGGGUCGCGUCGGUGUGCGCAGAAGGGUCUAUGUGUGGGCCUGCCUGGAGCCGCCGACGGCUGUGC	274	294	+ [Positive strand]	12	3	−34.8

### 3.4 The presence of miRNA star strands

It is very difficult to identify the star strand (miRNA*) sequences from the library, as it will be degraded soon after being exported to the cytosol. However, our results revealed that ten *T. tabaci* miRNA* families (mir‐14, mir‐184, mir‐8, mir‐276, mir‐210, mir‐1, mir‐3477, mir‐71, mir‐13, and let‐7) were identified within the known miRNA category (Table 6). The expression values (number of reads) of all miRNA*s were lower than that of their corresponding miRNAs (Table 6). Among the miRNA* family, mir‐8 and mir‐276 families were having the highest abundance with 308 and 258 copies, respectively. Our results also indicated the presence of miRNA* sequences in four of our novel miRNAs such as tta‐miR‐N6, tta‐miR‐N7, tta‐miR‐N8, and tta‐miR‐N9, although the abundance was low (Table 5). The complete characteristic features of these miRNA* sequences and their corresponding pre‐miRNA*s have been given in Tables 5 and 6.

**Table 6:** Details of *Thrips tabaci* miRNA*s obtained from this study. Information regarding mature, star and precursor sequences, start and end position, orientation, expression values, MFE value and (A+U) content, etc. have been given

MicroRNA Family	Name of the miRNA*	miRNA	miRNA*	Hairpin sequence	Start	End	miRNA* Abundance	Minimum Free Energy(MFE)	Hairpin G/C%
mir‐14	tta‐tta‐miR*‐14	UCAGUCUUUUUCUCUCUCCUAU	GGGGAGAGAUAAGGGCUUUGGCU(5)	GGGGAGAGAUAAGGGCUUUGGCUCGAUUUUAAAGUCAGUCAGUCUUUUUCUCUCUCC	345	366	5	−26.7	45.614033
mir‐184	tta‐miR*‐184	UGGACGGAGAACUGAUAAGGG	CCUUGUCAUUCUCGUGUCCGGU(21)	CGCCUCCUUGUCAUUCUCGUGUCCGGUUGUGCAUUCAACUUACUGGACGGAGAACUGAUAAGGGCGCG	592	612	21	−33.5	54.411762
mir‐8	tta‐miR*‐8	UAAUACUGUCAGGUAAAGAUGUC	CAUCUUACCGGGCAGCAUUAGAC(308)	UCUGUUCACAUCUUACCGGGCAGCAUUAGACUUGGAUUGAUAGCCUCUAAUACUGUCAGGUAAAGAUGUCGUCAGA	3,662	3,684	308	−27.7	43.421055
mir‐276	tta‐miR*‐276	UAGGAACUUCAUACCGUGCUCU	UAGCGAGGUAUAGAGUUCCUACG(196) AGCGAGGUAUAGAGUUCCUACG(62)	UCCAGUAGCGAGGUAUAGAGUUCCUACGUGGUGUUGGGUACAGUAGGAACUUCAUACCGUGCUCUUGGA	4,052	4,073	258	−33.9	49.275364
mir‐210	tta‐miR*‐210	CUUGUGCGUGUGACAGCGGCU	AGCUGCUGGACACUGCACAAG(1) AGCUGCUGGACACUGCACAAGA(7)	AGCUGCUGGACACUGCACAAGAUUAGACUUUGGAAAACUCUUGUGCGUGUGACAGCGGCU	10,847	1,0867	8	−28.09	50
mir‐1	tta‐miR*‐1	UGGAAUGUAAAGAAGUAUGGAG	CCAUACUUCCUUGCUUCCCAU(7) CCAUACUUCCUUGCUUCCCAUA(4)	GUUCCAUACUUCCUUGCUUCCCAUAUUGCCAUUUGAAACUUAUGGAAUGUAAAGAAGUAUGGAGC	581	602	11	−24.86	38.46154
mir‐3477	tta‐miR*‐3477	UAAUCUCAUGUGGUAACUGUGA	UCAGGGUUCCGCGUGAGGUUG(1)	GUAAUCUCAUGUGGUAACUGUGAGUUGUACUUGUACCUCAGGGUUCCGCGUGAGGUUGC	5,735	5,756	1	−31.3	49.152542
mir‐71	tta‐miR*‐71	UCUCACUACCUUGUCUUUCAUG	UGAAAGACAUGGGUAGUGAGAU(19) UGAAAGACAUGGGUAGUGAGAUG(20)	GGGUGACGUGAAAGACAUGGGUAGUGAGAUGUUUGCUGCUGUACAUCUCACUACCUUGUCUUUCAUGUUGCUC	1,176	1,197	39	−47.1	46.575344
mir‐13	tta‐miR*‐13	UAUCACAGCCACUUUGAUGAGC	GCCAUCAAUACGGCUGUGAGAGC(64) CCAUCAAUACGGCUGUGAGAGC(17)	GAGGCUGGAGCCAUCAAUACGGCUGUGAGAGCGUGAAUUUGAUACCGUAUCACAGCCACUUUGAUGAGCUCUGGCUUC	1,427	1,449	81	−41	51.282055
let‐7	tta‐miR*‐let‐7	UGAGGUAGUAGGUUGUAUAGU	CUGUACAACUUGCUAACUUUC(2) CUGUACAACUUGCUAACUUUCC(4)	GCCGGGUUGAGGUAGUAGGUUGUAUAGUAAUGAACUACAACACUUGGGAGUACUGUACAACUUGCUAACUUUCCCUCGC	1,826	1,846	6	−31.9	45.56962

### 3.5 Identification of plant miRNA family in *Thrips tabaci* sRNA library

Interestingly, this study has identified mir‐9774 (Expression value 6), a plant microRNA family in our *T. tabaci* sRNA library (Table 3).

### 3.6 Phylogenetic analysis of *Thrips tabaci* miRNAs

**Figure 4:**
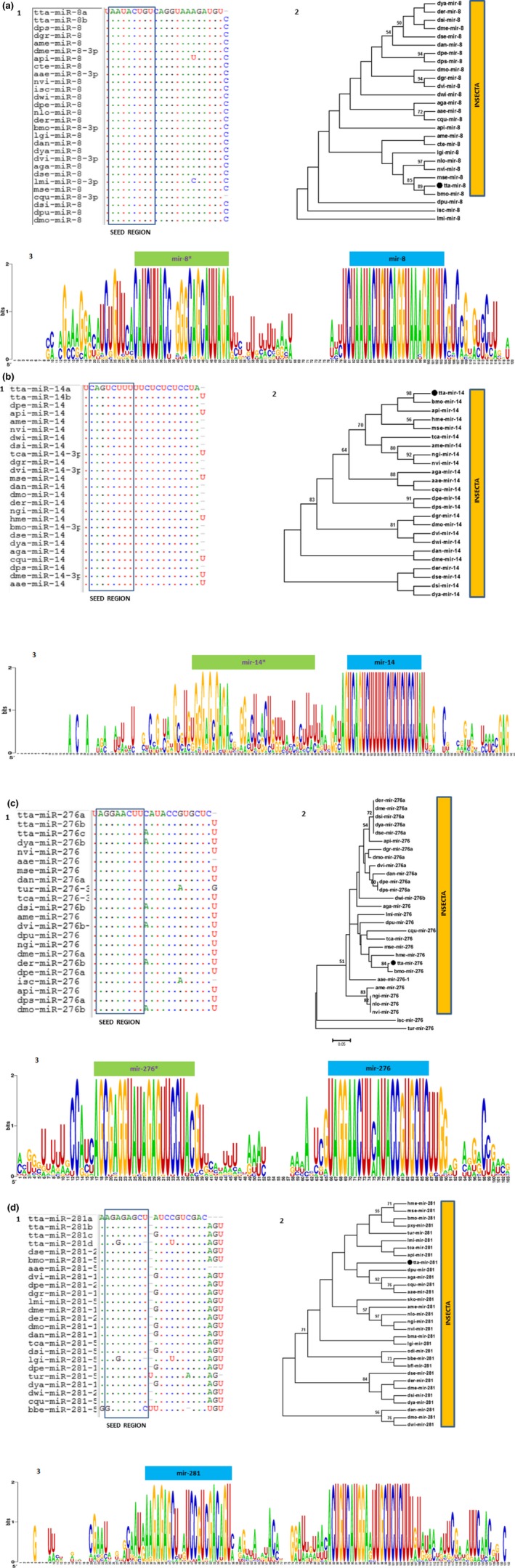
(a–d): 1. Homology in the seed region of the *Thrips tabaci* miRNAs (a–d are for mir‐8, mir‐14, mir‐276, and mir‐281, respectively) with respect to its counterpart from other insect species. The first three letters of each miRNAs indicating the name of the species (e.g.,: dya‐ *Drosophila yakuba*). (a–d): 2. Maximum Likelihood tree (RaxML.v.7.0.4) indicating the phylogenetic relationship of precursor miRNA sequences from various members of the animal kingdom. (a–d): 3. *Thrips tabaci* pre‐miRNAs weblogo indicating both mature (blue bar) and the star (green bars) sequences. Each logo consists of stacks of symbols, one for each nucleotide position in the sequence. The height indicates the sequence conservation at that nucleotide position and the height of symbols within the stack indicates the relative frequency of each nucleotide at that position

Phylogenetic analyses revealed that most of the known miRNAs are highly conserved (Table 4, Figure 4a1–d1 and Figure 4a3–d3) among various species within the Kingdom and the phylogenetic trees for miR‐8, miR‐14, miR‐276, and miR‐281 revealed that *T. tabaci* miRNAs grouped with the closely related species of insects (Figure 4a2–d2). Figure 4 also revealed that *T. tabaci* miRNAs are well conserved, particularly in the seed region compared to the homologous miRNAs from other species.

### 3.7 Identification of targets for *Thrips tabaci* miRNAs

Targets were predicted for known and novel miRNAs of *T. tabaci* employing miRanda with a scale of 0–7 to indicate the stringency of miRNA‐target pairing with the smaller numbers representing higher stringency. ESTs and transcriptome of *F. occidentalis* were used as a reference for target searches with a cut‐off score 140.

#### 3.7.1 Targets for known miRNAs from *Thrips tabaci*

**Figure 5:**
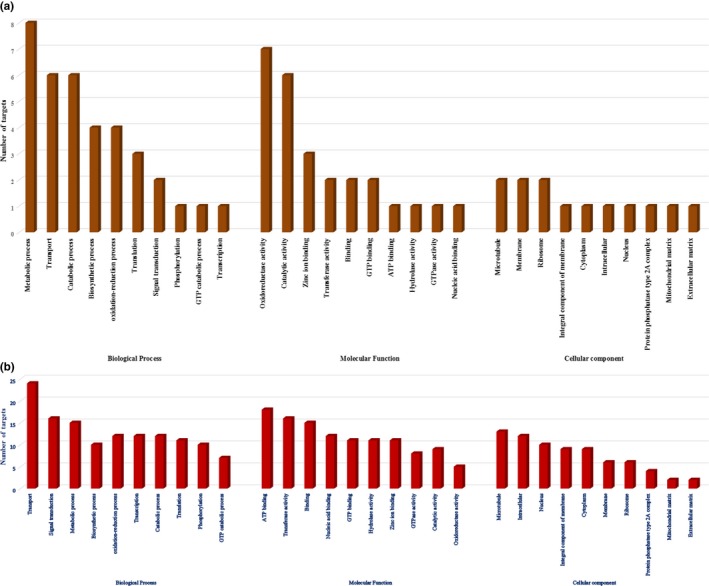
Gene Ontology (GO) classification of the putative target genes for the conserved *T. tabaci* miRNAs against ESTs (a) and transcriptome (b) sequences of *F. occidentalis*. GO terms was assigned to each target gene based on the annotation and were summarized into three main GO categories viz. (1) biological process (BP) (2) molecular function (MF), and (3) cellular component (CC). Only top ten subcategories are presented here

One hundred and thirty known miRNAs were searched for targets against ESTs and transcriptome sequences of *F. occidentalis*. A total of 218 and 1,025 targets were obtained from ESTs and transcriptome, respectively (Tables S1 and S2). The Blast‐2‐GO enrichment analysis was performed employing gene ontology (GO) terms for genes targeted by these miRNAs (Figure 5a,b). For those targets in the ESTs, three motifs were over‐represented in GO–BP (biological process) category *viz*. “metabolic process,” “transport,” and “catabolic process.” The GO–MF (molecular function) category was over‐represented by the motif “oxidoreductase activity” and “catalytic activity” (Figure 5a). On the other hand, GO terms enrichment analysis of miRNA targets in the transcriptome yielded motifs for “transport,” “signal transduction,” and “metabolic process” in GO‐BP category; while, GO‐MF category was over‐represented with motifs for “ATP binding,” “transferase activity,” and “binding” (Figure 5b). Complete details of the Blast‐2‐GO analysis were provided in Tables S3 and S4.

#### 3.7.2 Targets for novel miRNAs from *Thrips tabaci*

**Figure 6:**
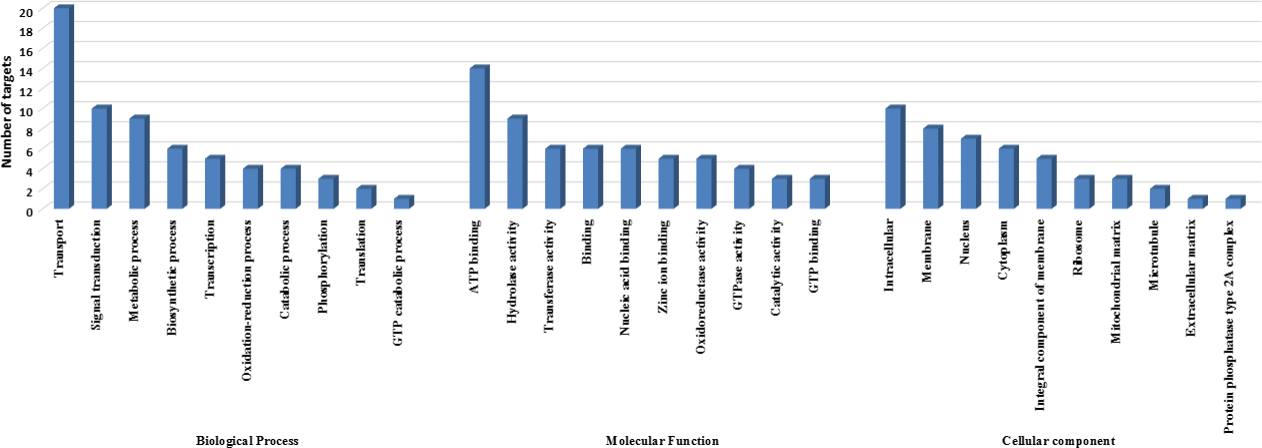
Gene Ontology (GO) classification of the putative target genes for the *T. tabaci* miRNAs against transcriptome sequences of *F. occidentalis*. GO terms was assigned to each target gene based on the annotation and were summarized into three main GO categories viz. (1) biological process (BP) (2) molecular function (MF), and (3) cellular component (CC). Only top ten subcategories are presented here

Novel miRNAs were searched for their targets in the *F. occidentalis* transcriptome. A total of 65 miRNA‐target pairs were obtained (Table S5), and further Blast‐2‐GO analysis indicated the over‐representation of “Transport” and “ATP binding” as GO‐BP and GO‐MF category, respectively (Figure 6 and Table S6).

#### 3.7.3 Synteny analysis using Circos

**Figure 7:**
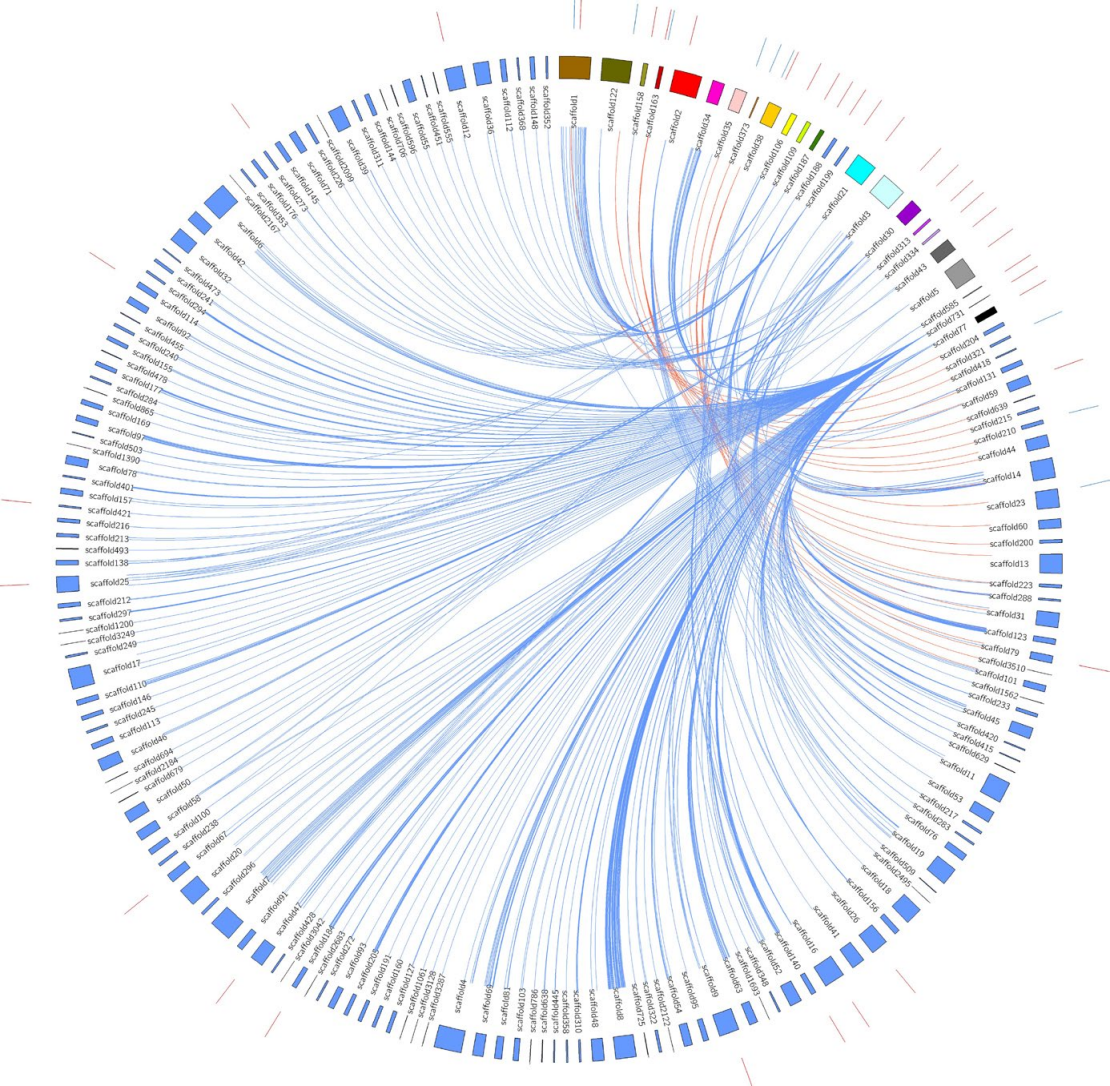
Map of the Western Flower Thrips, *F. occidentalis* scaffolds linking *T. tabaci* miRNAs and their putative targets prepared using Circos (Krzywinski et al., 2009). The outer circle represents the highlights of nine novel miRNA represented in blue and 34 known miRNA represented in red color. The inner circle marks each scaffold in a different color. The blue lines in the center of the figure connect a known miRNAs with its target that are represented across 173 scaffolds of *F. occidentalis* genome. Whereas, the orange lines in the center represent the interaction of novel miRNA with its target positions

The synteny analysis of the *T. tabaci* miRNAs and their targets were performed by employing circos (Krzywinski et al., 2009). In brief, the Blast analysis was performed using *T. tabaci* miRNA sequences (known and novel) against *F. occidentalis* scaffolds (Approx. largest 200). The positions of miRNAs were identified and their targets are represented in the Circos plot (Figure 7).

### 3.8 Validation of *Thrips tabaci* microRNAs

**Figure 8:**
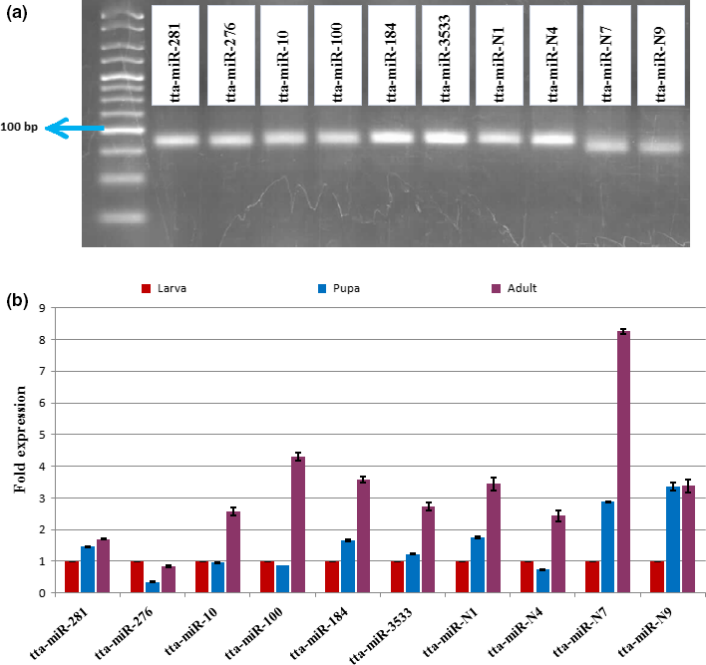
(a) Stem‐loop RT‐PCR analyses of six conserved and four novel miRNAs from *Thrips tabaci*. The products were resolved on 3% agarose gel in 1X TBE stained with ethidium bromide and HyperLadder^™^ 25 bp (Bioline, USA) used as marker. (b) Stem‐loop RT‐qPCR analysis of spatiotemporally expressed *T. tabaci* miRNAs in larva, pupa and adults. “*” and “**” means a statistically significant difference at level *p* < .05 and *p* < .001, respectively, for these miRNAs in the larva, pupae, and adult *T. tabaci*. The error bars indicate standard deviation for three biological replications

This study revealed 130 known and nine novel miRNAs from *T. tabaci*. However, further validation of these miRNAs was performed by (1) stem‐loop endpoint reverse transcriptase PCR (RT‐PCR) and (2) real‐time quantitative reverse transcriptase PCR (RT‐qPCR). Using stem‐loop endpoint RT‐PCR, we have validated six conserved *viz*. tta‐miR‐281, tta‐miR‐276, tta‐miR‐10, tta‐miR‐100, tta‐miR‐184, and tta‐miR‐3533 and four novel miRNAs *viz*. tta‐miR‐N1, tta‐miR‐N4, tta‐miR‐N7, tta‐miR‐N9 from *T. tabaci* using the primer sets as described (Table S7). All of these miRNAs were amplified with an approximate product size of 75 bp (Figure 8a).

Our study also quantified the expression level of the above‐mentioned ten miRNAs from *T. tabaci* larva, pupa, and adult using RT‐qPCR (Table S8, Figure 8b). Results suggested that the miRNA expression was higher in pupal and adult stages compared to larval stages in six microRNAs such as tta*‐*miR‐281, tta‐miR‐184, tta‐miR‐3533, tta‐miR‐N1, tta‐miR‐N7, and tta‐miR‐N9 (Figure 8b).

## 4 DISCUSSION

The onion thrips, *Thrips tabaci,* is an important pest species and a tospovirus vector causing significant negative impacts on yield and quality of various economically important crops (German et al., 1992). Although microRNAs are key gene regulators and are involved in many biological processes, including growth and development, no previous study has been conducted on the identification and validation of miRNAs in *T. tabaci*. MicroRNAs are known from more than 25 insect species, (Stark et al., 2007). Several miRNAs have been reported from various orders of insects such as Diptera, Hymenoptera, Coleoptera, Orthoptera, Lepidoptera, Hemiptera, Homoptera (Wu et al., 2013), and Thysanoptera (Rebijith, Asokan, Hande, & Krishna Kumar, 2016). This study reports the complete miRNA profile from onion thrips, *Thrips tabaci*. A small RNA library was prepared from the pooled samples of different developmental stages of *T. tabaci* and the high‐throughput Illumina deep*‐*sequencing technology (Avesson et al., 2012; Burnside et al., 2008; Ge et al., 2013; Koh et al., 2010) was used to identify miRNAs from the prepared library.

We used the *F. occidentalis* genome sequence as a reference for *T. tabaci*, as the complete genome *T. tabaci* is still not available in the database. The higher percentage of mapping (91%) was possible only because both these insects belong to the same family, Thripidae. Employing this approach, our study revealed 130 conserved and nine novel miRNAs from *T. tabaci*. The size distributions of the high‐quality reads were varied from 18 to 28 nts in our library and the peak was at the 25 nt*,* which was on par with previous studies (Ge et al., 2013; Liang, Feng, Zhou, & Gao, 2013; Sattar et al., 2012). Our study indicated the unique read distributes of 26–28 nts with a relative lower abundance, which is common in many small RNA libraries (Chang et al., 2016; Jagadeeswaran et al., 2010; Surridge et al., 2011; Zhang et al., 2013), indicating the presence of piRNAs. Piwi RNAs (piRNAs) are the class of small RNAs mediating chromatin modifications (Ross, Weiner, & Lin, 2014) which are derived mainly from retrotransposons and other repetitive elements with high sequence diversity (Ross et al., 2014; Siomi, Sato, Pezic, & Aravin, 2011; Zhang et al., 2013). Thus, our results indicated that *T. tabaci* genome not only harbors miRNAs but also other small RNAs such as piRNAs that might be involved in the transgenerational epigenetic inheritance (Weick & Miska, 2014).

MiRNAs are evolutionarily conserved regulators of gene expression (Rebijith et al., 2014; Zhang et al., 2009), and few can even act as markers in defining the evolutionary relationship in a wide range of insect species (Kakumani et al., 2015). Our homology and phylogeny analysis revealed that insect miRNAs are well‐conserved, despite considerable diversity in the genome (Figure 4a–d). MiRNA*s are not easily detectable as it degrades soon after being exported to the cytosol (Wu et al., 2013). However, our results indicated the presence of several miRNA*s (Tables 5 and 6) that matched to the same precursor sequences with their mismatched complementary mature miRNAs.

We identified the presence of a plant‐specific miRNA family, mir‐9774 in the *T. tabaci* sRNA library, and the same has been recently reported from *Triticum aestivum* L. and *Brachypodium distachyon* (L.) Beauv (Wei et al., 2009). Previous miRNA studies on cotton/melon aphid, *A. gossypii* also reported six plant miRNA family (Sattar et al., 2012). They also showed that such microRNAs were transformed into the aphid tissues (especially in gut contents) during the phloem sap ingestion. However, none of those six have been identified in our sRNA library.

Our results showed that the highest expression is for tta‐miR‐276 with an expression value of 26,418. Very recent studies showed that miR‐276 expressed in the ovaries of female locusts mediates progeny egg‐hatching synchrony by upregulating its target *brahma* (*brm*), a transcription coactivator gene (He et al., 2016). Thus, it is plausible that miR‐276 enhances *brm* expression to promote developmental synchrony and provide insight into the regulation of developmental homeostasis in *T. tabaci*. The second highest expression is for miR‐281 with an expression value of 18,063 and might be involved in the development and metamorphosis of *T. tabaci* as recent studies showed that miR‐281 regulates the expression of *ecdysone receptor* (*EcR*) isoform B, in *Bombyx mori* (Jiang et al., 2013). Another interesting microRNA obtained in the current study was miR‐8, and it can target the Wingless signalling pathway to regulate secretion of yolk protein precursors by the female mosquito fat body and accumulation into the developing ovaries (Lucas et al., 2015, http://www.smartscitech.com/index.php/RD/article/view/815). Therefore, it is quite possible that miR‐8 may play a key role in the reproductive processes of *T. tabaci*. An insect‐specific miR‐14 was identified in *T. tabaci* with an expression value of 12,453 and studies on lepidopteran insects showed the antiapoptotic role of miR‐14 (Kumarswamy & Chandna, 2010). The rest of the species‐specific miRNAs identified in *T. tabaci* might play important role in insect‐specific features, such as metamorphosis, parthenogenesis, and biogenesis of pheromones (Zhang et al., 2007). Whereas, the other invertebrate‐ and vertebrate‐specific miRNAs (Table 3) identified from *T. tabaci* required special attention, as their nonexistence in other species of insects could be due to the absence of complete genomic information for most of those insects (Ge et al., 2013).

The expression profile of miRNA varies spatiotemporally among different developmental stages (Li, Cassidy, Reinke, Fischboeck, & Carthew, 2009; Xu, Zhou, Wang, Auersperg, & Peng, 2006), and the developmental expression profiles (larval, pupal and adult stage) of ten microRNAs were studied by RT‐ qPCR (Figure 8b). The higher expression of tta*‐*miR‐281, tta‐miR‐184, tta‐miR‐3533, tta‐miR‐N1, tta‐miR‐N7, and tta‐miR‐N9 in *T. tabaci* pupal and adult stages reflected their possible role in parthenogenesis, adult development, and sexual reproduction. The high levels of miR‐276 in the larval stage indicated their possible involvement in insect‐specific features such as metamorphosis.

miRNAs regulate the gene expression through targeting transcripts that can bring about mRNA cleavage, mRNA decay or translational repression of target mRNAs by binding to 3′ UTRs, 5′ UTRs, and even to coding regions (Lytle, Yario, & Steitz, 2007). Thus, it is important to identify the gene targets and thereby we can understand the biological role of a particular miRNA. As miRNA targets have been identified using the (1) expressed sequence tags (ESTs) and (2) transcriptomic sequences of *F. occidentalis*. The GO annotations for the predicted targets were classified as potential biological process, cellular component, and molecular function. The putative targeted genes included signal transduction pathways, transcription factors, reproduction, embryo development, insect molting, immune response, and even metabolism. Overall, the results from our study indicated that these conserved and novel miRNAs identified from *T. tabaci* might play crucial regulatory role in the regulation of thrips growth and development.

## 5 CONCLUSIONS

In summary, the result from our study add to the pool of miRNA databases and is the first report of small RNAs from *T. tabaci*, a nonmodel insect lacking genome information. One hundred and thirty conserved and nine novel miRNAs were identified with high confidence and sufficient evidence is the major contribution of our study. Sequence analyses revealed that most of the *T. tabaci* miRNAs are highly conserved in various species, making miRNAs, a hallmark of evolutionarily conserved regulators of gene expression. To harmonize the data and to provide more useful biological insights, we have also carried out in silico analysis of identifying potential targets for these miRNAs. Our results indicated that the list of putative mRNA targets was very extensive and most of the putative target genes for *T. tabaci* miRNAs were associated with several KEGG pathways such as metabolic process, transport, translation, signal pathways, and oxidative phosphorylation. However, further experiments are required for the validation of these targets. Expression levels of *T. tabaci* miRNAs were validated by RT‐qPCR, and the results indicated few of these miRNAs have been predicted in the adult development process, which can be further utilized in gene functional studies through RNAi‐based approach or in developing miRNA mimics both for feeding and *in planta* expression (Agrawal, Sachdev, Rodrigues, Sowjanya Sree, & Bhatnagar, 2013; Jayachandran, Hussain, & Asgari, 2013; Nandety et al., 2015) as novel pest management strategies based on gene silencing and insect transgenesis.

## DATA AVAILABILITY

All relevant data are within the paper and its Supporting Information files. The small RNA Sequence data has been submitted to NCBI under the BioSample project ‘PRJNA350618’; BioSample Accession: ‘SAMN05943039’.

## CONFLICT OF INTEREST

The authors have declared that no competing interests exist.

## AUTHORS’ CONTRIBUTIONS

Conceptualization: KBR HRH, Experiments: KBR HRH, Reagents/materials: KBR RA SG, Writing—original draft: KBR, Writing—review and editing: KBR HRH RA SG NKK.
