# Supplementary figures and images for: Genome‐wide identification, expression profiling, and target gene analysis of microRNAs in the Onion thrips, Thrips tabaci Lindeman (Thysanoptera: Thripidae), vectors of tospoviruses (Bunyaviridae)
Source: Ecol Evol. 2018 Jun 7;8(13):6399–419. doi: 10.1002/ece3.3762 (PMC6053560; doi:10.1002/ece3.3762)

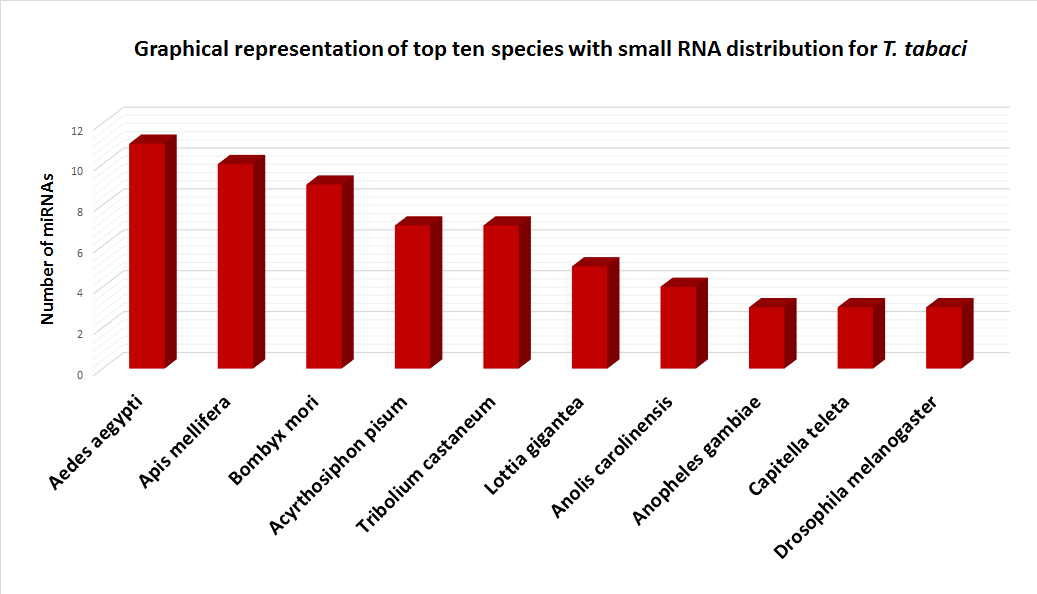

Supplement: Supplementary file 1 [file ECE3-8-6399-s001.tif]
