# Supplementary material for: Genome‐wide identification, expression profiling, and target gene analysis of microRNAs in the Onion thrips, Thrips tabaci Lindeman (Thysanoptera: Thripidae), vectors of tospoviruses (Bunyaviridae)
Source: Ecol Evol. 2018 Jun 7;8(13):6399–419. doi: 10.1002/ece3.3762 (PMC6053560; doi:10.1002/ece3.3762)
Supplement: Supplementary file 8 [file ECE3-8-6399-s008.docx]

Suppl. Table 7. List of universal reverse primer, stem-loop RT primers and forward primers employed in *T. tabaci* miRNA validation.

| **Sl.**  **No.** | **Oligo Name** | **Oligo Sequence ( 5' to 3')** |
| --- | --- | --- |
| 1 | Universal Reverse | ATCCAGTGCAGGGTCCGAGG |
| 2 | RT/tta-miR-281 | GTCGTATCCAGTGCAGGGTCCGAGGTATTCGCACTGGATACGACGTCGAC |
| 3 | F/tta-miR-281 | GCGGCGGAAGAGAGCTATCCGT |
| 4 | RT/tta-miR-276 | GTCGTATCCAGTGCAGGGTCCGAGGTATTCGCACTGGATACGACGAGCAC |
| 5 | F/tta-miR-276 | GCGGCGGTAGGAACTTCATACC |
| 6 | RT/tta-miR-10 | GTCGTATCCAGTGCAGGGTCCGAGGTATTCGCACTGGATACGACACAAAT |
| 7 | F/tta-miR-10 | GCGGCGGACCCTGTAGATCCGA |
| 8 | RT/tta-miR-100 | GTCGTATCCAGTGCAGGGTCCGAGGTATTCGCACTGGATACGACACAAGT |
| 9 | F/tta-miR-100 | GCGGCGGAACCCGTAGATCCGA |
| 10 | RT/tta-miR-184 | GTCGTATCCAGTGCAGGGTCCGAGGTATTCGCACTGGATACGACCCCTTA |
| 11 | F/tta-miR-184 | GCGGCGGTGGACGGAGAACTGAT |
| 12 | RT/tta-miR-3533 | GTCGTATCCAGTGCAGGGTCCGAGGTATTCGCACTGGATACGACATGTCC |
| 13 | F/tta-miR-3533 | GCGGCGGATGAAGTGTGACGTG |
| 14 | RT/tta-miR-N1 | GTCGTATCCAGTGCAGGGTCCGAGGTATTCGCACTGGATACGACTGGCCT |
| 15 | F/tta-miR-N1 | GCGGCGGAGGTAACTAACTTGCA |
| 16 | RT/tta-miR-N4 | GTCGTATCCAGTGCAGGGTCCGAGGTATTCGCACTGGATACGACAGACGA |
| 17 | F/tta-miR-N4 | GCGGCGGTGACTAGACTCTCACT |
| 18 | RT/tta-miR-N7 | GTCGTATCCAGTGCAGGGTCCGAGGTATTCGCACTGGATACGACCGCGCT |
| 19 | F/tta-miR-N7 | GCGGCGGTCAGGTACCAGAAGT |
| 20 | RT/tta-miR-N9 | GTCGTATCCAGTGCAGGGTCCGAGGTATTCGCACTGGATACGACCCTTCT |
| 21 | F/tta-miR-N9 | GCGGCGGCGCGTCGGTGTGCG |
