# Supplementary material for: Genome‐wide identification, expression profiling, and target gene analysis of microRNAs in the Onion thrips, Thrips tabaci Lindeman (Thysanoptera: Thripidae), vectors of tospoviruses (Bunyaviridae)
Source: Ecol Evol. 2018 Jun 7;8(13):6399–419. doi: 10.1002/ece3.3762 (PMC6053560; doi:10.1002/ece3.3762)
Supplement: Supplementary file 9 [file ECE3-8-6399-s009.docx]

Suppl. Table 8. *T. tabaci* microRNA specific primers employed in the RT-qPCR.

| **miRNA** | **Sequence (5' - 3')** |
| --- | --- |
| tta-miR-281 Forward | AAGAGAGCTATCCGTCGAC |
| tta-miR-276 Forward | TAGGAACTTCATACCGTGCTC |
| tta-miR-10 Forward | ACCCTGTAGATCCGAATTTGT |
| tta-miR-100 Forward | AACCCGTAGATCCGAACTTGT |
| tta-miR-184 Forward | TGGACGGAGAACTGATAAGGG |
| tta-miR-3533 Forward | ATGAAGTGTGACGTGGACAT |
| tta-miR-N1 Forward | AGGTAACTAACTTGCAGGCCA |
| tta-miR-N4 Forward | TGACTAGACTCTCACTCGTCT |
| tta-miR-N7 Forward | TCAGGTACCAGAAGTAGCGCG |
| tta-miR-N9 Forward | CGCGTCGGTGTGCGCAGAAGG |
