# Supplementary material for: Genome‐wide identification, expression profiling, and target gene analysis of microRNAs in the Onion thrips, Thrips tabaci Lindeman (Thysanoptera: Thripidae), vectors of tospoviruses (Bunyaviridae)
Source: Ecol Evol. 2018 Jun 7;8(13):6399–419. doi: 10.1002/ece3.3762 (PMC6053560; doi:10.1002/ece3.3762)
Supplement: Supplementary file 10 [file ECE3-8-6399-s010.docx]

S1 Figure. Graphical representation of top ten species with small RNA distribution for *T. tabaci*

S1 Table. Potential targets for the identified known miRNAs of *T. tabaci* with EST orthologs of *F. occidentalis*.

S2 Table. Potential targets for the identified known miRNAs of *T. tabaci* with the transcriptomic sequences of *F. occidentalis*.

[S3 Table.](http://journals.plos.org/plosone/article/asset?unique&id=info:doi/10.1371/journal.pone.0163635.s004)Complete Functional categories of gene ontology classification of the putative target genes for *T*. *tabaci* miRNAs against ESTs of *F*. *occidentalis*.

### [S4 Table.](http://journals.plos.org/plosone/article/asset?unique&id=info:doi/10.1371/journal.pone.0163635.s005)Complete Functional categories of gene ontology classification of the putative target genes for the known miRNAs of *T. tabaci* against transcriptome sequences of F.occidentalis.

### [S5 Table.](http://journals.plos.org/plosone/article/asset?unique&id=info:doi/10.1371/journal.pone.0163635.s006)Potential targets for the identified novel miRNAs of *T. tabaci* with Transcriptome sequences of F. occidentalis.

### [S6 Table.](http://journals.plos.org/plosone/article/asset?unique&id=info:doi/10.1371/journal.pone.0163635.s007)Complete Functional categories of gene ontology classification of the putative target genes for the novel miRNAs of *T. tabaci* against transcriptome sequences of F.occidentalis.
